# Anti-Inflammatory Therapy in Diabetic Retinopathy

**DOI:** 10.1155/2014/947896

**Published:** 2014-02-23

**Authors:** Katarzyna Zorena

**Affiliations:** Department of Clinical and Experimental Endocrinology, Institute of Maritime and Tropical Medicine, Medical University of Gdańsk, Powstania Styczniowego 9b, 81-519 Gdynia, Poland

The concequence of type 1 (T1DM) and type 2 diabetes mellitus (T2DM) is the development of chronic vascular complications encompassing micro- as well as macrocirculation. Diabetic retinopathy (DR), microvascular and visually devastating diabetic complication, is estimated to be the leading cause of blindness in working-aged adults in the developed countries, and this upward trend is continuing. Retinal vascular leakage, inflammation, and neovascularization (NV) are the main features of DR. Also, closely linked to retinopathy are neural changes such as neuronal cell death, gliosis, microglial activation, and altered glutamate metabolism. Gaining knowledge of the mechanisms of action of factors influencing retinal neovascularization, as well as the search for new, effective treatment methods, especially in advanced stages of DR, puts special importance on research concentrating on various treatment methods of diabetic retinopathy [1–3].

This special issue encompasses 16 papers including original scientific theses and clinical studies which deepen our current knowledge by presenting additional DR risk factors. Apart from this, it presents review papers widening our knowledge on new, noninvasive diagnostic methods of testing for diabetic maculopathy as well as on new trends in the treatment of late vascular complications in adults and young patients with T1DM and T2DM. The first step in managing DR is to reduce the risk of its development and progression by controlling the risk factors such as hyperglycemia, hypertension, and hyperlipidemia. Therefore, in the study presented by J. Tołwińska et al. the authors prove that intensification of insulin therapy in the form of continuous subcutaneous insulin infusion (CSII) might have a beneficial effect on reducing the risk of development of early micro- and macrovascular damage in young patients with T1DM. They find a significant correlation between the arterial intima-media thickness (IMT) and flow-mediated dilatation (FMD) with the metabolic control of diabetes.

In another paper K. Semeran et al. evaluated the bioelectric function of the retina and the serum levels of interleukin-17 (IL-17), vascular endothelial growth factor (VEGF), and adrenomedullin (ADM) in young patients with T1DM. Interestingly, even before recognizing the fundus eye changes characteristic for DR, the authors had observed bioelectric retinal disturbances in adolescent patients with T1DM. The authors suggest that the presented results provide new possibilities in diagnosing preclinical chronic diabetic complications in T1DM patients. The paper by K. Zorena et al. presents the relationship between serum transforming growth factor beta 1 (TGF-*β*1) concentrations and the duration of T1DM in children and adolescents. The authors show that serum TGF-*β*1 concentrations and the duration of the disease are independent risk factors of microangiopathy development in children and adolescents with T1DM.

Meanwhile B. Urban et al. present the results of endothelial cell density (ECD) and central corneal thickness (CCT) in adolescent T1DM patients. They discover that the mean density of corneal endothelium cells in patients with diabetes was reduced by 18% in comparison to the control group. In addition, the researchers have shown a significant correlation between corneal endothelium density and the duration of diabetes in patients with T1DM.

Two interesting review papers by K. Zorena et al. and D. Raczyńska et al. included in this special issue discuss the newest data on biomarkers involved in the risk of development and progression of DR in patients with T1DM and T2DM and current trends in the monitoring and treatment of DR in young adults.

An exquisite review paper by B. L. Sikorski et al. provides knowledge on the new technique the Optical Coherence Tomography (OCT). OCT is used by the ophthalmologists to obtain the images of retinal cross-section in patients with diabetes. The traditional approach to the diagnosis of DM includes fundus ophthalmoscopy and fluorescein angiography. Although they're very useful clinically, these methods do not contribute much to the evaluation of retinal morphology and its thickness. That is why OCT facilitates the measurement of the macular thickening, quantification of the diabetic macular oedema, and the detection of vitreoretinal traction.

Due to its high prevalence, incidence, and risk of macrovascular and microvascular complications, T2DM is one of the potentially most damaging diseases and the biggest public health problems at present. Diabetic eye disease with its complications, especially diabetic retinopathy that leads to macular edema and retinal neovascularization, is the leading cause of visual dysfunction and blindness among adult working-age population in economically developed societies worldwide. In this special issue M. Tomic et al. and S. Kaštelan et al. have proven in their original papers that apart from other known DR risk factors it is also the adipose tissue that markedly contributes to the chronic, low-grade inflammation in T2DM patients. The authors examined the correlation between the inflammatory and clotting markers, endothelial dysfunction markers, and the anthropometric parameters as well as their connection with DR in T2DM patients. Their review paper included in the same issue presents the most up-to-date knowledge on the pharmacological treatment in diabetic retinopathy [S. Kaštelan et al.].

Glucagon-like peptide-1 (GLP-1) is a potent glucoincretin hormone released from intestinal L-cells in response to the nutrient ingestion. An emerging clinical interest in the management of T2DM has been shown for GLP-1. In vitro studies presented by A. Puddu et al. showed that GLP-1/GLP-1R axis might potentially contribute to the prevention of retinal pigment epithelium (RPE) cell dysfunction and DR.

The paper of A. Gębka et al. presents an original research on the effect of oral supplementation with alpha-lipoic acid (ALA) in patients with T1DM and T2DM. The authors show that oral supplementation with ALA at a relatively low dose (300 mg once daily) for 3 months maintains functional vision in T1DM patients and even improves it in T2DM patients. Authors suggest that supplementation with ALA represents an achievable adjunct therapy to help prevent loss of vision in patients with T1DM and T2DM.

This special issue also presents data on the genetic studies on the DR. The aim of the study by J. Wang et al. was to investigate whether polymorphisms of the two genes in the complement pathway, complement factor H (CFH) and complement factor B (CFB), are associated with DR in patients with T2DM. The authors report that CFH-rs800292 and CFB-rs1048709 polymorphisms are associated with the development of DR as well as with its delayed progression. However, the D. Romaniuk et al. in their preliminary study present the results of gene expression of insulin-like growth factor 1 (IGF1), insulin-like growth factor 1 receptor (IGF1R), and insulin-like growth factors binding protein-3 (IGFBP3) in epiretinal membranes (ERMs) of patients with PDR. They suggest That IGF 1 and IGF1R differential expression can be Involved in the pathogenesis or progression of proliferative vitreoretinal disorders.

Subsequent studies were presented by A. M. Abu El-Asrar et al. The authors investigated the correlations between the levels of brain-derived neurotrophic factor (BDNF) and the levels of high-mobility group box-1 (HMGB1) in the vitreous fluid and serum of patients with PDR and in the retinae of rats with diabetes. They also investigated the effect of intravitreal administration of HMGB1. The authors show that blocking HMGB1 signaling pathways might be a novel therapeutic strategy for neuronal dysfunction in vision-threatening diabetic retinopathy.

Finally, G. Mohammad et al. investigated the hypothesis that poly-(ADP-ribose) polymerase (PARP) activation mediates retinal neuropathy by reducing brain-derived neurotrophic factor (BDNF), synaptophysin, and glutamine synthetase (GS) levels in the retina. To test this hypothesis, they measured the levels of reactive oxygen species (ROS) generation, PARP, phosphorylated ERK1/2 (p-ERK1/2), BDNF, synaptophysin, GS, and cleaved caspase-3 in the retina of diabetic rats. In addition, the authors analyzed whether treatment with the PARP inhibitor 1,5-isoquinolinediol suppresses the neurodegenerative changes in the retinas of diabetic rats. They reported a beneficial effect of the PARP inhibitor in increasing neurotrophic support and ameliorating early retinal neuropathy induced by diabetes.

Summarizing, all the data presented and discussed by all the authors who have contributed to this special issue point to the new diagnostic as well as therapeutic perspectives of DR.
